# The Mechanism of Icariin in Regulating BMSCs for Treating Osteoporosis: A Review

**DOI:** 10.2174/0118715303467650260722042115

**Published:** 2026-07-28

**Authors:** Chengjie Wang, Zhenyu Wu, Yawei Xu, Yuyi Li, Zhenwei Man, Kang Liu, Jian Wang, Wenlin Lin, Fengqing Qie, Xiaolin Shi, Yifeng Yuan

**Affiliations:** 1 The Second School of Clinical Medicine, Zhejiang Chinese Medical University, Hangzhou, Zhejiang Province, China;; 2 The Second Affiliated Hospital of Zhejiang Chinese Medicine University, Hangzhou, Zhejiang Province, China;; 3 Jinhua Academy of Zhejiang Chinese Medical University, Hangzhou, Zhejiang Province, China;; 4 Ruian Hospital of Traditional Chinese Medicine, Ruian, Zhejiang Province, China;; 5 Yushi Health Research Institute, Tokyo, Japan

**Keywords:** Icariin, bone marrow mesenchymal stem cells, osteoporosis, traditional chinese medicine, skeletal disease, Epimedium

## Abstract

**Introduction:**

Osteoporosis (OP) is a common skeletal disease mainly caused by the imbalance between bone formation and bone resorption. Dysfunction and senescence of bone marrow mesenchymal stem cells (BMSCs) are critical pathogenic factors for OP, resulting in impaired osteogenic differentiation, increased adipogenic shift, and enhanced susceptibility to inflammation and oxidative stress. Therefore, the purpose of this review is to systematically synthesize current evidence on how Icariin (ICA)—a major bioactive flavonoid from *Epimedium*—exhibits therapeutic potential against OP by targeting BMSC senescence and regulating multiple underlying signaling networks.

**Methods:**

This narrative review systematically synthesized preclinical and mechanistic evidence regarding Icariin-mediated regulation of BMSC function in osteoporosis. Relevant cellular, animal, pharmacological, and mechanistic studies were reviewed, with emphasis on BMSC senescence, osteogenic/adipogenic lineage commitment, oxidative stress, inflammation, and major signaling pathways, including Wnt/β-catenin, Notch, BMP/Runx2/Osx, MAPK, and OPG/RANK/RANKL.

**Results:**

ICA can promote osteogenic differentiation and inhibit osteoclastogenesis by regulating multiple signaling pathways simultaneously. ICA activates the Wnt/β-catenin pathway to enhance the stability of β-catenin and its downstream transcriptional programs, modulates Notch signaling through regulating the activity of DLL1, Jagged1, and NICD, and promotes the BMP/Runx2/Osx axis, which favors osteoblast lineage commitment. Additionally, it controls the activity of MAPK (ERK, p38, JNK) and restores bone homeostasis by managing the OPG/RANK/RANKL system, leading to reduced osteoclastogenesis and decreased production of inflammatory cytokines. In addition to regulating lineage commitment, ICA alleviates BMSC senescence by reducing oxidative stress, DNA damage, mitochondrial dysfunction, and SASP-associated microenvironmental deterioration.

**Discussion:**

Collectively, these effects promote osteogenesis, maintain stem cell vitality, and mitigate OP-induced bone loss. The hierarchy, crosstalk, and cell-type specificity of ICA-targeted signaling networks require further elucidation to facilitate clinical translation.

**Conclusion:**

Preclinical evidence suggests ICA is a promising natural compound with the potential to mitigate OP progression by comprehensively regulating BMSC functions and bone remodeling pathways. Future research needs to focus on rigorous clinical trials and single-cell-level studies to determine the specific BMSC subpopulations responsive to ICA and optimize its therapeutic application.

## 1 INTRODUCTION

Osteoporosis (OP) is a systemic skeletal disease that features low bone mineral density and microarchitectural deterioration, both of which increase the susceptibility to fractures [1]. From a public health perspective, OP has become one of the most common chronic diseases among middle-aged and older adults because of its high prevalence, substantial disease burden, and fracture-related disability [2]. Epidemiological data also indicate the scale of this issue. Based on a national survey conducted recently, the general prevalence of OP among people aged 50 and over is 19.2%, with a significantly higher prevalence among females (32.1%) compared to males (6.9%). Among people aged 65 and older, the prevalence increases to 32.0%, and the difference between genders becomes even larger [3]. These findings highlight the urgent need to develop more effective strategies for the prevention and treatment of OP, particularly in elderly populations and high-risk women. Although the exact pathogenesis of OP remains partially elucidated, current evidence suggests a multifactorial etiology encompassing genetic predisposition, hormonal imbalances, nutritional deficiencies, and lifestyle factors [4]. In clinical practice, standard anti-osteoporotic therapy consists of basic calcium and vitamin D supplementation, agents that inhibit bone resorption, and drugs that promote bone formation. Although these treatments reduce fracture risk, long-term use may also be associated with adverse outcomes, including an increased risk of atypical femoral fractures [5]. Therefore, the search for safer and more efficient new therapeutic methods has become a key part of modern research into OP.

BMSC dysfunction is considered a key pathogenic mechanism underlying the development and progression of OP [6]. In OP, BMSCs exhibit multiple functional impairments: their osteogenic differentiation capacity is impaired, whereas their adipogenic differentiation tendency is enhanced [7-10]. Concurrently, the migration and homing abilities are weakened, cellular senescence accelerates, and apoptosis increases [11]; all these changes collectively exacerbate OP progression.

*Epimedium* (*Berberidaceae* family), a traditional Chinese medicine, has been extensively applied to prevent and treat various disorders such as OP, tendon and joint diseases, cardiovascular diseases, and menstrual irregularities [12-14]. Icariin (ICA) is the main active ingredient in *Epimedium*, and it has gained much attention from recent pharmacological research. Experimental evidence indicates that ICA can regulate BMSC lineage commitment by promoting osteogenic differentiation and indirectly suppressing osteoclastogenesis. This effect is associated with activation of the Wnt/β-catenin and BMP-2/Runx2/Osx signaling pathways [15]. Meanwhile, ICA also blocks important adipogenic regulators such as PPARγ to reduce the formation of adipocytes [16]. Furthermore, ICA exhibits immunomodulatory and anti-inflammatory effects that can reduce chronic inflammation in bone tissue and thus decrease bone loss and structural damage [17]. ICA has demonstrated therapeutic potential for the treatment of OP, but the exact mechanisms behind its anti-osteoporotic effects have yet to be fully understood.

While several recent reviews [18, 19] have discussed the general anti-osteoporotic effects of ICA, most primarily focus on its direct regulation of mature osteoblasts or osteoclasts. The novel perspective of this review lies in shifting the focus upstream to the bone marrow microenvironment, systematically synthesizing how ICA rescues BMSC senescence. By integrating fragmented mechanisms—specifically, how ICA mitigates ROS accumulation, DNA damage, and telomere attrition while coordinating multiple signaling pathways (Wnt/β-catenin, Notch, BMP/Smad, and MAPK) to prevent adipogenic shift—this study provides a comprehensive multi-target framework. Consequently, this review contributes to a deeper understanding of ICA's pharmacological profile and provides a theoretical framework for the future translational development of ICA in OP.

## 2 ICARIIN

*Epimedium*, also called Xianlingpi, was first recorded in Shen Nong Ben Cao Jing (Divine Farmer's Materia Medica), which is one of the oldest Chinese materia medica texts. Traditional Chinese Medicine categorizes it as a kidney tonifying herb that strengthens Yang, dispels wind, and expels dampness, and has been used for a long time to treat kidney deficiency and bone and joint problems [20]. Modern pharmacological studies show that *Epimedium* has a complex and diverse chemical composition with many bioactive substances and a broad spectrum of biological activities. It has been proven to have anti-inflammatory, immunomodulatory, antitumor, and neuroprotective effects, indicating possible therapeutic applications for different pathological states such as immune-mediated diseases, cardiovascular and cerebrovascular diseases [21], skeletal metabolic anomalies, and nervous system injuries [22]. To date, approximately 270 chemical constituents have been identified and recognized in *Epimedium*, mostly flavonoids, polysaccharides, lignans, and alkaloids [23]. ICA, the major prenylated flavonol glycoside extracted from *Epimedium*, shows distinct pharmacological activities closely related to its unique chemical structure (C_33_H_40_O_15_, molecular weight 676.66 g/mol) [24]. Biochemically, ICA possesses a core flavonoid skeleton modified by a prenyl group at the C-8 position, which significantly enhances its lipophilicity and cell membrane permeability compared to other conventional flavonoids [25]. This specific lipophilic structural feature not only facilitates its intracellular uptake but also contributes to its extensive biological effects, such as regulating bone metabolic balance, adjusting lipid metabolism, reducing inflammation, and having some degree of antitumor activity.

ICA maintains the balance between bone formation and bone resorption, thereby preventing OP pathogenesis. Bone remodeling occurs when there is a constant state of new bone formation (osteoblasts) and bone resorption [26] (osteoclasts). Accumulating evidence indicates that ICA can promote the differentiation and proliferation of osteoblasts, increase the activity of Alkaline Phosphatase (ALP) [27], which is an important indicator of osteoblast function, thereby promoting the mineralization of the bone matrix. Meanwhile, ICA inhibits osteoclast maturation and resorptive activity, keeping the balance of bone turnover. This dual mechanism of stimulating osteogenesis while inhibiting bone loss may be critical for slowing the progression of OP. At the cellular level, ICA has been shown to promote the proliferation and differentiation of human osteoblasts, raise the levels of osteogenesis-related proteins, and improve the cells’ resistance to oxidative stress. Under hypoxic conditions, ICA reduces apoptosis and maintains the viability of rat calvarial osteoblasts [28]. Furthermore, ICA activates the STAT3 signaling pathway, resulting in increased transcription of the downstream osteogenic gene osteocalcin (Ocn), leading to more bone formation and protection against alveolar bone loss due to estrogen deficiency [29]. Osteoblasts can undergo transdifferentiation into adipocytes [30], and notably, ICA inhibits this adipogenic shift, thereby maintaining a pool of mature osteoblasts for bone remodeling. On the molecular level, ICA affects some of the signaling pathways related to bone metabolism, including Wnt/β-catenin, MAPK, PI3K/Akt, and ERα/RANK [31]. These pathways are important for maintaining bone homeostasis. Activating the osteogenic pathways of Wnt/β-catenin and modulating other pathways such as MAPK and PI3K/Akt, ICA promotes osteoblast differentiation, and the modulation of ERα/RANK signaling by ICA results in the inhibition of osteoclast formation. By coordinating the regulation of all those signaling pathways, ICA ultimately improves bone remodeling and mitigates osteoporosis-related bone loss [32].

OP is closely associated with chronic low-grade inflammation. Senescent bone marrow macrophages (S-MΦs) participate in the formation of OP by secreting a large number of pro-inflammatory cytokines and creating a long-term local inflammatory environment [33]. Although cellular autophagy exerts anti-senescence effects, its specific role in modulating inflammation in the context of OP treatment remains elusive. A previous study demonstrated that ICA promotes autophagy, significantly attenuates the inflammatory response of S-MΦs, and restores the osteogenic capacity of senescent bone marrow-derived mesenchymal stem cells (S-BMSCs), which aids in mitigating bone loss in OP mice [34]. Additionally, ICA attenuated OP-induced pathological alterations by stimulating chondrocyte autophagy, lowering apoptosis, and reducing SASP factor expression [35]. According to this result, another study indicates that ICA could block the activation of some significant inflammatory mediators, including NF-κB, reduce the production of inflammatory cytokines, attenuate inflammatory damage to bones, and have a powerful osteoprotective impact [36].

## 3 BMSCS AND OP

### 3.1 Biological Functions of BMSCs

BMSCs originate from the mesoderm and are multipotent stem cells capable of self-renewal and differentiation into multiple lineages [37]. They are crucial for skeletal development, postnatal bone growth, and bone repair [38]. BMSCs are widely distributed and can be isolated from various anatomical sites, such as the bone marrow, fat tissue, placenta, and umbilical cord [39]. Under suitable circumstances, they can differentiate into various lineages, such as adipocytes, osteoblasts, and chondrocytes [40]. The differentiation fate of BMSCs is tightly regulated by key transcription factors [41]. For instance, the combined action of CCAAT/enhancer-binding protein alpha (C/EBPα) and peroxisome proliferator-activated receptor gamma (PPARγ) pushes BMSCs towards the adipogenic pathway, while the transcription factor RUNX2 promotes their commitment to the osteoblastic pathway [42]. In contrast, under the control of SOX9, BMSCs tend to become chondrocytes [42]. This intricately regulated transcriptional network dictates lineage commitment and is fundamental to bone remodeling and the pathogenesis of OP.

BMSCs serve as essential progenitor cells for bone formation, so the number and function of BMSCs are crucial for maintaining bone homeostasis. Accumulating evidence indicates that the participation of BMSCs in bone formation relies on multiple signaling pathways acting in concert, with the Wnt pathway and bone morphogenetic protein (BMP) signaling playing especially important roles in connecting bone resorption and bone formation [43]. BMP family members such as BMP2 can promote bone formation by activating the Smad1/5/8 signal transduction pathway, thus promoting osteoblast differentiation and increasing the expression of key osteogenic transcription factors such as RUNX2 [44]. At the same time, the Wnt signaling pathway participates in the regulation of bone metabolism through stabilizing β-catenin, which is necessary for maintaining the stability of the skeleton [45]. Activation of the Wnt/β-catenin signaling pathway not only increases the transcriptional activity of RUNX2, but also affects the expression of bone resorption-related factors in the bone marrow microenvironment [46]. Consequently, the synergistic interplay between Wnt and BMP signaling ensures a tight coupling of bone formation and resorption, thereby modulating BMSC osteogenesis and overall bone remodeling.

### 3.2 Mechanisms Associated with BMSCs Senescence

Cellular senescence refers to a state of irreversible growth arrest with a significant reduction in the ability to proliferate [47]. The senescence of BMSCs is controlled by many related mechanisms, such as the shortening of telomeres, mitochondrial malfunction, and the accumulation of DNA damage [48]. Importantly, BMSC senescence should not be viewed as a simple linear sequence of events. From a systems biology perspective, these mechanisms form a highly interconnected signaling network characterized by intensive crosstalk. For instance, excessive intracellular ROS not only directly induces DNA double-strand breaks but also accelerates telomere shortening. Conversely, severe telomere dysfunction triggers a profound DNA damage response (DDR) and disrupts mitochondrial homeostasis, leading to further ROS overproduction [49]. This continuous positive feedback loop creates a vicious cycle that synergistically and irreversibly drives BMSCs into senescence. Thus, effective pharmacological interventions must target this integrated network rather than isolated pathways. As BMSCs become senescent, their ability to differentiate into osteoblasts is substantially reduced [50]. Many studies show that BMSCs senescence is closely linked to the development of skeletal metabolic disorders, such as OP [51]. For instance, in aged rat models, not only is bone metabolism imbalanced, but also BMSCs obtained from the bone marrow of aged rats show typical characteristics of senescence, such as increased expression of senescence-related genes (such as p16), obvious mitochondrial shrinkage [52]. Similarly, BMSCs from OP model rats exhibit severely impaired osteogenic differentiation capability and upregulated SASP, along with severe mitochondrial dysfunction [53]. Inducing BMSCs senescence experimentally by means of DNA damage or telomere depletion can result in a decrease in the bone-forming ability and an increase in osteoclast-mediated bone resorption, thus accelerating the development of OP [54]. All these results point to the conclusion that BMSCs senescence is an important pathological process leading to the disturbance of bone remodeling and the imbalance of bone metabolism seen in OP.

#### 3.2.1 Free Radical Theory

Under normal physiological circumstances, there is a delicate equilibrium between the body's antioxidant protection mechanism, which includes antioxidant enzymes and non-enzyme antioxidants, and the formation and removal of free radicals. Aging or exposure to pro-aging stimuli can disrupt this balance, resulting in a reduction of the body's internal antioxidant capacity and an increase in the accumulation of free radicals [55]. The resulting oxidative stress causes extensive damage to cellular structures, especially the mitochondria. This damage also disrupts metabolic pathways (*e.g*., glucose and lipid metabolism), thereby exacerbating age-related pathologies, such as OP [56].

BMSCs’ growth and osteogenic differentiation capability is largely determined by the level of oxidative stress inside the cells. Generally speaking, higher levels of reactive oxygen species (ROS) greatly reduce the proliferative activity of BMSCs, cause them to differentiate towards the adipogenic lineage, and decrease their ability to differentiate into bone tissue. High-ROS environments also harm the immunoregulatory functions of BMSCs and increase the expression of senescence-related phenotypes [57]. One study investigated the cell cycle dynamics of senescent BMSCs, and revealed that BMSCs from a premature-aging mouse model exhibited G0/G1 cell cycle arrest and significantly elevated intracellular ROS levels, along with changes in the amount of important immune-related molecules [58]. On the contrary, another study demonstrated that when excessive intracellular ROS was scavenged, the proliferative and migratory abilities of BMSCs would be recovered. Several experimental studies have also shown that antioxidant compounds from traditional Chinese medicine can increase the osteogenic differentiation of BMSCs through reducing the level of ROS. For example, resveratrol can inhibit the generation of ROS inside cells by activating the AMPK signal pathway, which can attenuate the senescence process of BMSCs [59]. Furthermore, it is worth noting that NAD⁺ and the NAD⁺/NADH ratio are important for controlling redox reactions; in senescent BMSCs, there is an abnormal increase in NAD⁺ levels and an increased NAD⁺/NADH ratio, and exogenous NAD⁺ supplementation greatly restores their osteogenic differentiation ability [60]. Collectively, these findings suggest that restoring redox homeostasis represents a potential strategy to mitigate BMSC senescence and metabolic dysfunction.

#### 3.2.2 DNA Damage

The progressive accumulation of DNA damage, coupled with age-related declines in DNA repair efficacy, accelerates cellular senescence. Cells are subjected to endogenous or exogenous stress, and their double-stranded DNA structures may be broken, causing single- or double-strand breaks [61]. These lesions activate the cell's repair mechanisms and trigger a signaling cascade known as the DNA damage response (DDR). Histone H2AX, one of the main mediators of DDR, is quickly phosphorylated on a C-terminal serine residue following DNA double-strand breaks to generate γ-H2AX, which accumulates at DNA damage sites. Therefore, H2AX is essential for initiating DNA repair and coordinating multiple repair factors [62].

Studies show that γ-H2AX expression is greatly enhanced in senescent BMSCs [63]. Persistent DNA damage signals cause BMSCs to enter premature senescence, which is marked by a halt in DNA synthesis, an increase in senescence-related proteins such as p21 and p16, higher levels of β-galactosidase activity, and a significant decrease in their ability to undergo osteogenic differentiation [64].

#### 3.2.3 Telomeres and Telomerase

Telomeres are special structures made up of nucleoproteins found at the ends of eukaryotic chromosomes that consist of tandem TTAGGG repeat sequences along with their respective binding proteins. Their main function is to keep the ends of chromosomes stable and prevent end-to-end fusion and degradation [65]. During constant cell division, telomeres progressively shorten; when they reach a certain length, the cells will stop dividing and enter a state of replicative senescence, where their ability to divide decreases dramatically. Telomerase can combat this ongoing telomere shortening by adding telomeric repeats, thus maintaining telomere length and conferring replicative immortality. In BMSCs, the loss of telomerase function has been proven to decrease the capacity for proliferation as well as change the differentiation potential of the cells, making them more prone to differentiating into adipocytes or fibroblasts instead of osteoblasts [66]. This might be because of problems with telomeres causing damage to mitochondria and disrupting cellular energy metabolism, both of which accelerate BMSC senescence [67]. Additionally, specific mutations in genes encoding telomerase complex components are associated with the development of metabolic bone diseases such as OP [68]. Collectively, this evidence implies that the abnormal telomerase function of BMSCs is one of the important regulatory mechanisms for cell aging and thus for the occurrence and development of OP.

In addition to telomere- and telomerase-related mechanisms described above, BMSCs senescence is also regulated by a range of epigenetic and molecular factors. These include altered expression of aging-associated genes [69], such as SIRT family deacetylases, Klotho, p16, p21, and p53, as well as epigenetic modifications involving DNA methylation, histone modification, and noncoding RNAs (*e.g*., miRNAs). Together, these regulatory networks reshape the transcriptional and functional landscape of BMSCs, thereby contributing to cellular senescence, impaired osteogenic potential, and bone metabolic imbalance. A comprehensive schematic overview of the interconnected pathophysiological network, illustrating how the vicious cycle of BMSC senescence drives lineage shift and promotes OP, is presented in Fig. (**1**).

### 3.3 Mechanisms of BMSCs Senescence in the Pathogenesis of OP

#### 3.3.1 BMSCs Senescence Leads to a Decline in Osteoblast Differentiation Ability

Under appropriate inductive conditions, BMSCs have the capacity to differentiate into osteoblasts. However, this osteogenic potential progressively declines with advancing age. Functionally, aging BMSCs exhibit a reduced proliferation rate, diminished commitment toward the osteoblastic lineage, and a restricted capacity for self-renewal. Experimental studies also show that senescent mesenchymal stem cells tend to differentiate into adipocytes rather than osteoblasts, which leads to insufficient bone formation [70].

Reduced osteogenic differentiation of BMSCs is one of the major causes of the decrease in bone formation with age. This process is influenced by numerous intracellular and extracellular factors, such as increased oxidative stress, chronic low-grade inflammation, and activation of the SASP [70]. These factors can affect osteogenic commitment by changing several signaling pathways, such as Wnt/β-catenin, BMP-Smad, and Notch, which can disturb the gene expression programs needed for osteogenic differentiation and therefore hinder bone formation.

#### 3.3.2 BMSCs Senescence Promotes Osteoclastogenesis

Osteoclasts (OCs)’ development and function are controlled by a variety of cytokines. Among them, receptor activator of nuclear factor κB ligand (RANKL), interleukin-6 (IL-6), tumor necrosis factor-α (TNF-α), granulocyte-macrophage colony-stimulating factor (GM-CSF), and osteoprotegerin (OPG) all play important roles in regulating the proliferation, differentiation, and activation of osteoclast precursors *via* different signaling pathways [71]. BMSCs are closely involved in the regulation of these key cytokines, particularly the RANKL-RANK-OPG system. RANKL is a negative regulator of osteogenic differentiation in BMSCs and also has an inhibitory effect on osteogenesis-induced bone formation. RANKL binds to its receptor RANK on osteoclast precursor cells and promotes osteoclast differentiation and maturation and inhibits osteoclast apoptosis.

Under physiological conditions, BMSCs secrete OPG, a soluble decoy receptor that competes with RANKL for binding to RANK, thus preventing RANKL from interacting with RANK and effectively inhibiting osteoclastogenesis. However, as BMSCs undergo senescence, they downregulate OPG expression, impairing its capacity to neutralize RANKL, which consequently leads to excessive osteoclastogenesis and hyperactivity [72]. Consequently, BMSCs senescence disrupts the normal regulation of the RANKL-RANK-OPG axis, promoting osteoclastogenesis and bone resorption, leading to the progression of OP.

## 4 ICA REGULATES RELATED SIGNALING PATHWAYS AFFECTING OSTEOGENIC DIFFERENTIATION OF BMSCS

### 4.1 ICA Regulates the Wnt/β-catenin Signaling Pathway

The Wnt/β-catenin signaling pathway is a central regulatory axis in bone metabolism, and there is accumulating evidence that this pathway is disrupted during the development of many bone-related diseases, especially OP [73]. In the canonical Wnt pathway, Wnt ligands bind to Frizzled receptors and the co-receptors LRP5/6 at the cell surface to form a ternary complex, thereby initiating a downstream signaling cascade [74]. Activated Dsh proteins then inhibit the APC-Axin-GSK-3β degradation complex that usually causes β-catenin phosphorylation and degradation by the proteasome. Consequently, β-catenin is stabilized and accumulates in the cytoplasm. Stabilized β-catenin moves into the nucleus and associates with TCF/LEF family transcription factors to activate the transcription of target genes, such as c-myc and cyclin D1 [75]. This transcriptional program promotes osteoblast differentiation and maturation, and inhibits osteoblast apoptosis, promoting bone formation.

In addition to this canonical pathway, other Wnt proteins can also signal *via* non-canonical pathways such as the Wnt/Ca^2+^ pathway, PCP pathway, and PKA pathway [76], all of which participate in the fine-tuning of bone remodeling. Interestingly, bioactive elements from TCM “*Epimedium”* have been observed to activate Wnt/ β-catenin signaling through increasing β-catenin expression, leading to osteogenic differentiation of BMSCs and suggesting a potential therapeutic strategy for the prevention and treatment of OP. In general, the Wnt/ β-catenin pathway acts as a major molecular hub controlling the equilibrium between bone formation and resorption, and it is a vital target for understanding OP pathogenesis and creating new drug therapies [77].

As reported in previous studies, it has been demonstrated that ICA, which is one of the main active flavonoids contained in *Epimedium*, has strong osteogenic effects mainly *via* the Wnt/β-catenin signaling pathway. Studies show that ICA can increase important proteins such as β-catenin and c-myc by activating this pathway, thereby enhancing BMSC proliferation and osteogenic differentiation, offering therapeutic potential for OP. Similarly, total flavonoids from *Epimedium* were reported to activate the same pathway, increasing the expression levels of β-catenin and cyclin D1 mRNA and effectively promoting the osteogenic differentiation of BMSCs, thus providing an additional candidate strategy for OP prevention and therapy [78]. *In vitro* experiments further demonstrated that adding ICA at concentrations ranging from 0.15 to 15.00 μmol/L to cultures of osteoblasts isolated from rat mandibles significantly enhanced osteoblast proliferation and differentiation after 72 h, accompanied by upregulation of β-catenin transcript levels, suggesting that ICA may strengthen osteogenic differentiation and bone-forming capacity of mandibular osteoblasts *via* modulation of the Wnt/β-catenin pathway [79]. Consistently, ICA at concentrations of 5-40 μmol/L significantly increased the expression of osteogenic markers such as bone sialoprotein II and Runt-related transcription factor 2 (Runx2) through activation of Wnt/β-catenin signaling in human femur-derived BMSCs [80]. In a complementary study, BMSCs from OP model rats were cultured in ICA-containing osteogenic medium, and immunohistochemistry showed that cells in the ICA-treated group exhibited the lowest degree of senescence, along with enhanced migratory capacity and osteogenic differentiation; moreover, β-catenin mRNA expression was markedly higher than in the control group [81].

Taken together, these findings indicate that the pro-osteogenic effects of ICA on BMSCs and osteoblasts are largely dependent on regulation of the Wnt/β-catenin signaling pathway, involving stabilization of β-catenin protein and transcriptional activation of downstream target genes such as c-myc and Cyclin D1.

### 4.2 ICA Regulates Notch Signaling Pathway

The Notch pathway is a widely expressed and highly conserved cell signaling system in both vertebrates and invertebrates, and it plays a crucial role in regulating fundamental biological processes, including cell proliferation, differentiation, and programmed cell death through contact-dependent communication between neighboring cells [82]. In mammals, this pathway comprises four single-pass transmembrane receptors (Notch1-4) and five ligands (Jagged1, Jagged2, DLL1, DLL3, and DLL4), together with core components such as CSL and DSL family proteins, regulatory cofactors, and other associated molecules [83]. Unlike many classical signaling cascades, activation of the Notch pathway does not depend on secondary messengers. Upon ligand binding to the extracellular domain of a Notch receptor, the receptor undergoes sequential proteolytic cleavage by TACE and γ-secretase, releasing the Notch intracellular domain (NICD). NICD then moves into the cell’s nucleus, where it interacts with a transcription regulator called CSL/CBF-1 and recruits coactivators, thereby upregulating downstream target genes (coactivators), including those from the HES, HEY, and HERP groups [84]. Accumulating evidence suggests that Notch signaling has an important regulatory effect on the osteogenic differentiation of BMSCs, affecting their lineage commitment and thus participating in bone remodeling and the pathophysiology of OP [85].

In a rat model of OP induced by corticosterone combined with ovariectomy, *Epimedium* glycosides were shown to activate the Notch signaling pathway by increasing the expression of Delta-like ligand 1 (DLL1) and TACE (ADAM17) [86]. Activation of Notch signaling promoted osteogenic differentiation of BMSCs and increased the secretion of osteogenesis-related factors such as osteocalcin, type I collagen, and Runx2 in ovariectomized rats, directing BMSCs toward the osteoblastic lineage and producing a strong osteoprotective effect during OP progression. Another study showed that ICA treatment reduced the expression of Jagged1 on the surface of adipocytes inside the bone marrow cavity [87]. Jagged1, an important ligand of the Notch pathway, participates in the signal activation process during the early stage of cartilage formation, and Jagged1-mediated Notch signaling generally encourages the transformation of MSCs into adipocytes and restricts their osteogenic potential. These findings suggest that ICA may reduce the adipogenic shift of BMSCs by reducing the expression of adipogenesis-related factors. Similarly, ICA greatly enhanced the expression of osteogenesis-related genes and improved ALP activity in MC_3_T_3_-E_1_ and C_3_H_10_T_1/2_ cells [88]. In an ovariectomized mouse model, the mRNA levels of major Notch pathway components were elevated, and ICA intervention substantially reduced their expression. Collectively, these findings indicate that ICA can promote osteoblast differentiation *in vitro* and improve estrogen-deficiency-induced OP *in vivo*, partly by suppressing excessive Notch signaling.

In summary, evidence suggests that ICA modulates BMSC proliferation and lineage specification by targeting key components of the Notch signaling pathway (Jagged1, Notch1, CBF-1) to prevent OP pathogenesis. The Notch pathway is notable because it acts as a bidirectional regulator of bone remodeling, and its specific effects are highly dependent on the cellular microenvironment, ligand-receptor combinations, and temporal activation. ICA likely modulates this complex regulatory network through a multi-target and context-dependent manner.

### 4.3 ICA Modulation of the BMP/Runx2/Osx Signaling Pathway

The BMP/Runx2/Osx signaling pathway plays an important role in the differentiation of osteoblasts and maintaining the homeostasis of bone metabolism, bone formation, and bone repair [89]. Bone morphogenetic proteins (BMPs) belong to the TGF-β superfamily and are powerful promoters of bone formation; they can cause BMSCs to differentiate into different lineages, such as chondrocytes, osteoblasts, and dentin-forming cells [90]. Runx2, a runt-related transcription factor, is a main transcriptional regulator of osteoblast-lineage cells and drives the maturation of osteoblast precursors [91]. Osterix (Osx), a downstream effector of Runx2, also regulates the expression of osteogenesis-specific functional proteins and promotes the osteogenic differentiation of BMSCs [92]. When this pathway is activated, BMP ligands bind to and form heteromeric complexes of BMP type I and type II receptors on the cell surface, thereby initiating the intracellular Smad signaling cascade through phosphorylation. Activated R-Smads associate with Co-Smads to form transcriptional complexes that enter the nucleus and bind to osteoblast-specific cis-acting elements (OSEs) to initiate the transcription of Runx2 and its downstream target Osx. A series of molecular events take place leading to the elevation of several osteogenic markers, including osteoblastin, type I collagen, and osteocalcin, and enhancing intramembranous and endochondral bone formation, which contributes to the anti-osteoporotic effect of BMP/Runx2/Osx signaling [93].

Studies indicate that total flavonoids from *Epimedium* can boost the expression of osteogenesis-related markers such as type I collagen, osteocalcin, and osteoblastin *via* the activation of the BMP2/Runx2/Osx signaling pathway. Activation enhances ALP activity, forming mineralized calcium nodules, promoting the osteogenic commitment of BMSCs and accelerating new bone formation, and slowing down the deterioration of OP [94]. *In vitro* and *in vivo* studies have shown that both ICA and BMP-2 can promote the proliferation of osteoblasts and the formation of bone in animals with bone defects. Mechanistically, ICA increases the activity of the BMP-2/Smad4/Runx2 pathway and promotes osteoblast development and intracellular calcium accumulation by enhancing the expression of osteogenic genes such as Osx, Runx2, and ALP, thereby promoting bone formation [95]. In a Sprague-Dawley rat fracture model, ICA activated the BMP-2/Smad5/Runx2 signaling axis, resulting in osteogenic proliferation and differentiation of BMSCs [96]. Consistently, the osteogenic effects of ICA were further confirmed to be dependent on BMP signaling and its downstream Smad proteins [97].

Taken together, current evidence indicates that ICA exerts its pro-osteogenic and anti-osteoporotic effects primarily by modulating the BMP/Runx2/Osx signaling pathway. By activating key factors such as BMPs, Runx2, and Osterix, ICA promotes the expression of osteogenic genes—including osteocalcin, type I collagen, and bone-bridging proteins—and induces the directed differentiation of BMSCs into osteoblasts, while also providing additional antioxidant protection to bone tissue.

### 4.4 ICA-regulated MAPK Signaling Pathway

Mitogen-activated protein kinases (MAPKs) are a family of serine/threonine protein kinases widely expressed in eukaryotic cells and serve as key mediators transmitting extracellular signals to the nucleus. The MAPK family comprises three major subfamilies: extracellular signal-regulated kinases 1/2 (ERK1/2), c-Jun N-terminal kinases (JNK), and p38 MAPKs. Among these, the ERK pathway, as a classical signaling cascade, is mainly involved in regulating cell proliferation and differentiation and plays an important role in maintaining osteoclast function [98]. The p38 MAPK subfamily, which includes four isoforms (p38 α, β, γ, and δ), promotes the osteogenic differentiation of BMSCs upon activation, whereas the JNK pathway is crucial for the regulation of osteoblast activity [99].

Extracellular stimuli lead to MAPK signaling activation, generally going through a three-step phosphorylation process: MAP3K is initially activated, followed by the phosphorylation and activation of MAP2K; then MAP2K phosphorylates and activates MAPK. Activated MAPKs can enhance the expression of important osteogenic transcription factors such as Runx2 and increase the phosphorylation level of Smad1/5/8 proteins, thereby converting extracellular signals into intracellular responses and working together to promote osteogenic differentiation and bone formation [100]. Previous studies have shown that ICA can activate the MAPK signaling pathway, promoting the osteogenic differentiation of BMSCs, which provides an important mechanism for the use of ICA to treat OP.

Studies show that ICA can promote the osteogenic differentiation of BMSCs mainly by activating and finely regulating the MAPK signaling pathway. Recent studies [101] indicated that ICA activates the ERK and p38 MAPK pathways, leading to an increase in downstream transcription factors such as ELK1 and c-Myc, which significantly enhances the proliferative ability of rat BMSCs, providing a new target for OP treatment. Similarly, ICA significantly increased ALP activity *via* MAPK components’ (ERK, p38, JNK) phosphorylation and promoted expression of osteogenic marker genes (type I collagen, osteocalcin, osteoblastin), thus promoting osteogenic differentiation of BMSCs [102]. This study also indicated that ICA accelerates bone matrix formation by activating ERK signaling and increasing p38 MAPK activity. In terms of bone metabolic homeostasis, ICA at 1 μM was reported to suppress JNK pathway activity, reducing the pro-apoptotic impact of the JNK pathway on BMSCs and maintaining their osteogenic potential [103]. Taken together, all these findings suggest that the pro-osteogenic effects of ICA on BMSCs are associated with the accurate regulation of the MAPK pathway by ICA. By adjusting both ERK and p38 MAPK as well as JNK pathways along with their downstream effectors such as ELK1 and c-Myc, ICA changes the expression pattern of osteogenic genes and finally exerts a strong anti-osteoporotic effect.

### 4.5 ICA Regulates the OPG/RANK/RANKL Signaling Pathway

Osteoprotegerin (OPG) is a key regulator of the OPG/RANK/RANKL signaling axis [104]. Acting as a decoy receptor, OPG competes with RANKL for binding sites on the surface of osteoclast precursors, thus preventing RANKL from binding to RANK. This competitive binding interrupts downstream signaling cascades, thereby suppressing osteoclastogenesis and attenuating bone resorption.

OPG, RANK, and RANKL are the core components constituting the OPG/RANKL/RANK signaling axis, which is necessary to maintain the stability of bone metabolism [105, 106]. Accumulating evidence indicates that this regulatory system plays an important role in bone remodeling *via* multiple signaling pathways [107]. Firstly, in the NF-κB pathway, RANKL binds to RANK to recruit TRAF6, then it activates NIK, facilitating the nuclear translocation of the NF-κB complex [108]. This process increases the amount of c-Fos and works together with NFAT to promote the transcription of osteoclast-specific genes. Secondly, the AKT pathway: RANK binds to TRAF6, and PI3K is activated by TRAF6, and then PI3K promotes the activity of NF-κB and osteoclast differentiation [109]. Lastly, the JNK/MAPK pathway is activated upon RANK-TRAF6 interaction, causing ERK and JNK to get activated one after another. This improves JNK's activity, induces c-Jun phosphorylation, and facilitates the formation of the AP-1 complex (c-Jun/c-Fos), which facilitates the differentiation of osteoclast precursors into mature osteoclasts [110]. Fourth, the calcium-dependent calcineurin/NFATc1 pathway (Ca^2+^-CN-NFATc1) increases the transcription of many genes needed for osteoclast activity when it becomes active, thereby further exacerbating osteoclastogenesis [111]. In summary, elucidating the regulation of the OPG-RANK-RANKL signaling axis is crucial for developing effective strategies to prevent or reverse impaired bone microarchitecture and fragility fractures associated with OP.

BMSCs and osteoblasts produce and secrete OPG, maintaining the equilibrium of OPG and RANKL to coordinate the osteoblasts' bone-forming activity and osteoclasts' bone-resorbing action, thus achieving the homeostasis of the whole bone metabolism. ICA can enhance the expression of the OPG/RANKL system *via* an estrogen receptor-dependent route, subsequently inhibiting the maturation of osteoclasts and influencing the differentiation of osteoblasts [112]. Likewise, in a streptozotocin-induced diabetic OP rat model, ICA treatment could not only reduce blood glucose and increase BMD, but also decrease serum bone turnover markers [113]. Additionally, ICA increased the expression of Runx2 and the OPG/RANKL ratio, indicating that its anti-osteoporotic effect might be related to Runx2 and the OPG/RANKL signal pathway. Further investigation of the combination of ICA and mechanical stimulation in ovariectomized mice showed that applying both mechanical loading and ICA together led to a better increase in the OPG/RANKL ratio than using just one of these methods [114]. This suggests that their combined application may represent a synergistic therapeutic strategy for OP. Also, it has been observed that ICA can stop the release of pro-inflammatory cytokines such as TNF-α and IL-6, which generally cause bone resorption by increasing the expression of RANKL [115]. In summary, ICA restores the balance between bone formation and bone loss by modulating key components of the OPG-RANK-RANKL signal axis. Future research must further elucidate these underlying mechanisms to facilitate the development of novel targeted therapies for patients with OP. Fig. (**2**) illustrates the multi-target mechanisms by which ICA regulates BMSC senescence and restores bone homeostasis. To further facilitate mechanistic integration and improve reader understanding, Table **1** summarizes the principal signaling pathways involved in the regulatory effects of ICA on BMSC function and bone remodeling in osteoporosis.

### 4.6 Safety and Toxicology

A critical prerequisite for the clinical translation of any pharmacological agent is a comprehensive evaluation of its safety and toxicological profile. Current evidence suggests that ICA has a generally favorable safety profile, but long-term toxicological data and standardized dosing regimens remain insufficient. Human evidence remains limited; in a randomized, double-blind, placebo-controlled trial, orally administered ICA was generally well tolerated, with gastrointestinal discomfort reported mainly at the highest tested dose [116]. Meanwhile, ICA is characterized by poor aqueous solubility and limited oral bioavailability, which remain major barriers to its clinical translation [117]. Therefore, advanced delivery strategies, including ICA-loaded nanoplatforms and bone-targeting liposomes, have been explored to improve local bioavailability at bone remodeling sites and reduce systemic exposure [118]. Despite these promising safety characteristics, exhaustive long-term toxicological evaluations are still needed. In particular, future studies should clarify potential drug-drug interactions, including the modulation of intestinal UDP-glucuronosyltransferases (UGTs), in order to establish standardized and safe dosing regimens for the long-term management of osteoporosis.

### 4.7 Challenges in Clinical Translation and Limitations

While extensive *in vitro* and *in vivo* studies highlight the osteoprotective mechanisms of ICA, these preclinical findings must be interpreted within a cautionary framework before being extrapolated to clinical applications. First, animal models, such as ovariectomized rodents, do not perfectly replicate the complex pathophysiology and slow progression of human age-related osteoporosis. Second, the robust pharmacological effects observed in cellular models often require relatively high concentrations of ICA, which may be difficult to achieve *in vivo* due to its complex pharmacokinetic profile, limited aqueous solubility, and low oral bioavailability.

Furthermore, current literature lacks robust, large-scale, randomized controlled clinical trials to confirm the safety, optimal dosing regimen, and long-term efficacy of ICA in human populations. Future translational research should not only focus on rigorous clinical validation but also explore advanced targeted drug delivery systems—such as ICA-loaded nanomaterials, targeted liposomes, or bone-tissue engineering scaffolds—to enhance local bioavailability at the bone remodeling site while minimizing systemic off-target effects.

## CONCLUSION

From a network pharmacology framework, the pathogenesis of OP is driven by perturbations across a complex molecular network rather than a single defective pathway. Consequently, traditional single-target interventions often fail to fully restore bone homeostasis. The current review highlights that ICA acts as a potent pleiotropic agent, simultaneously modulating multiple critical network hubs—such as the Wnt/β-catenin, Notch, BMP/Smad, and MAPK signaling axes. By orchestrating these converging networks, ICA effectively uncouples the vicious cycle of BMSC senescence and provides a comprehensive, multi-target osteoprotective strategy. Current preclinical evidence suggests that ICA is a promising candidate for the treatment of OP. However, these preclinical findings must be interpreted with caution. Future rigorous clinical trials are warranted to validate the safety and efficacy of ICA in human subjects. Furthermore, single-cell technologies should be employed to elucidate the specific BMSC subpopulations responsive to ICA. In conclusion, ICA represents a promising natural therapeutic candidate for preventing and treating skeletal aging and OP.

## Figures and Tables

**Fig. (1):**
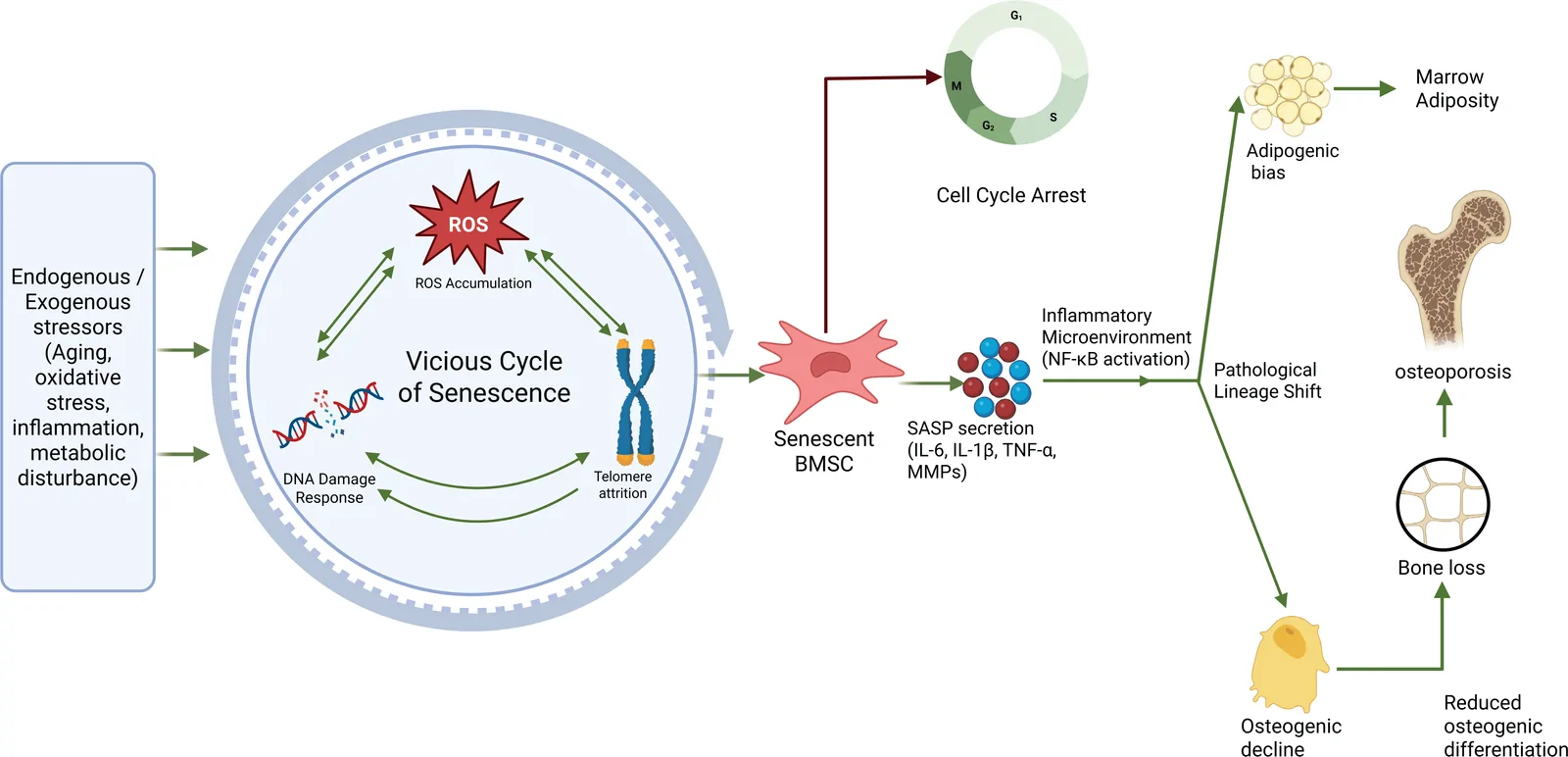
The interconnected pathophysiological landscape and vicious cycle of BMSC senescence in osteoporosis. This diagram illustrates the non-linear, reciprocal relationships between the primary drivers of BMSC senescence, including the accumulation of reactive oxygen species (ROS), the induction of DNA damage response (DDR), and telomere attrition. These factors forge a self-perpetuating feedback loop that creates a pro-senescence microenvironment characterized by mitochondrial dysfunction, enhanced senescence-associated secretory phenotype (SASP), and chronic low-grade inflammation. This pathologic milieu ultimately disrupts the lineage commitment of BMSCs, favoring adipogenic shift at the expense of osteogenic potential, thereby driving the development and progression of OP. Created in BioRender. wang, C. (2026) https://BioRender.com/dzctj2k.

**Fig. (2):**
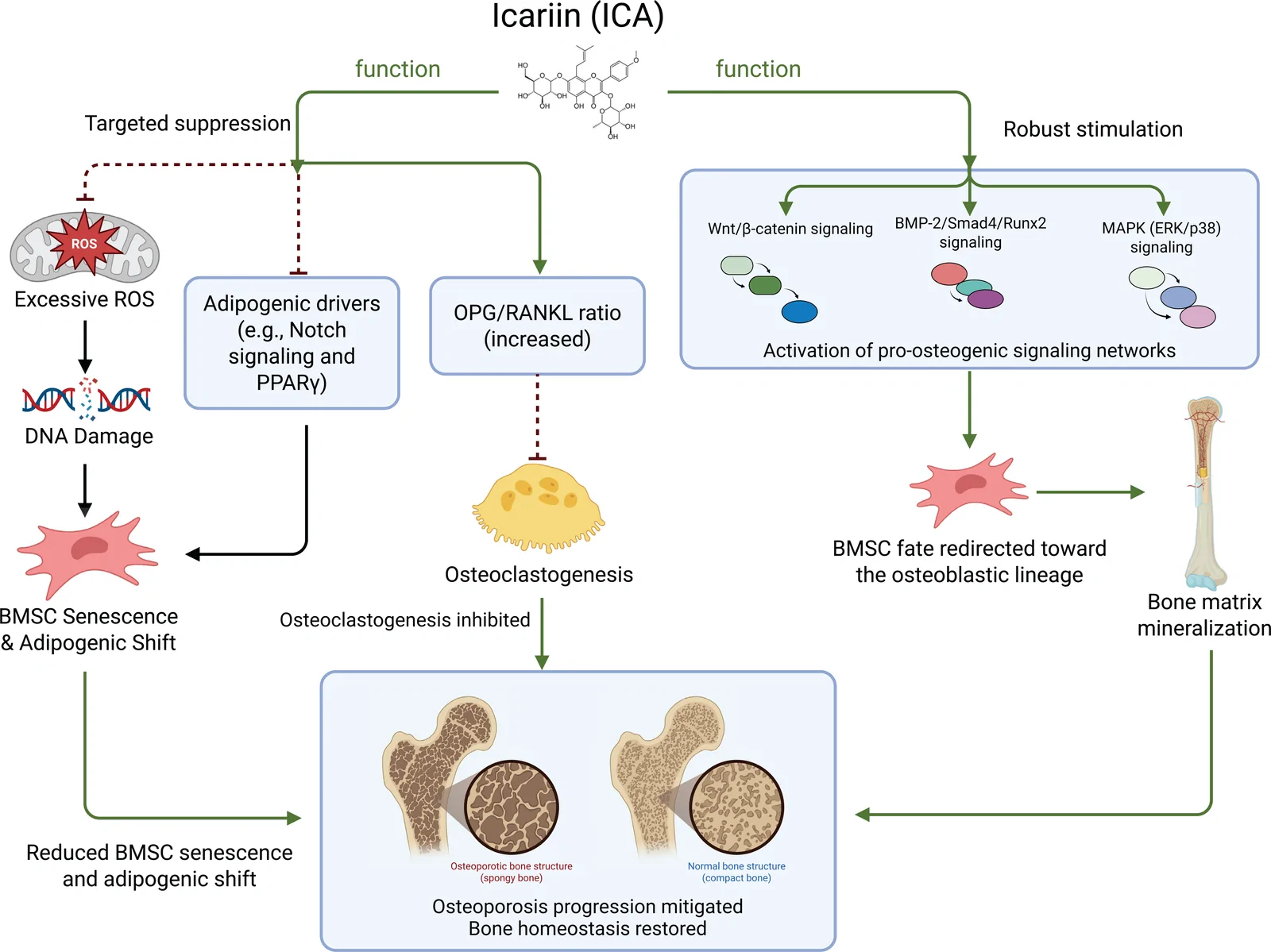
Comprehensive mechanistic network illustrating Icariin (ICA) as a multi-target pleiotropic regulator to rescue BMSC senescence and restore bone homeostasis. ICA acts as a potent orchestrator, simultaneously: activating the pro-osteogenic signaling axes (*e.g*., Wnt/β-catenin, BMP-2/Smad/Runx2, and MAPK pathways) to promote osteoblast differentiation and maturation; suppressing the pro-senescence triggers by scavenging ROS and inhibiting the DDR; and modulating the lineage fate by inhibiting Notch and PPARγ to prevent marrow adiposity. Furthermore, ICA increases the OPG/RANKL ratio to attenuate osteoclastogenesis. The convergence of these targeted networks results in uncoupling the vicious cycle of BMSC senescence and effectively restoring the delicate balance between bone formation and resorption. Created in BioRender. wang, C. (2026) https://BioRender.com/5rvshpn.

**Table 1:** Major signaling pathways involved in the regulatory effects of icariin on BMSCs and osteoporosis.

**Pathway**	**Major Molecular Targets**	**Regulatory Effect of Icariin**	**Biological Consequence in BMSCs / Bone Remodeling**	**Relevance to Osteoporosis**
Wnt/beta-catenin signaling	Wnt ligands, Frizzled, LRP5/6, Dsh, APC/Axin/GSK-3beta complex, beta-catenin, TCF/LEF, Runx2	Icariin activates canonical Wnt/beta-catenin signaling, stabilizes beta-catenin, and promotes its nuclear translocation and downstream osteogenic transcription.	Promotes BMSC proliferation and osteogenic differentiation, enhances osteoblast maturation, and suppresses apoptosis of osteoblast-lineage cells.	Improves osteogenic potential and helps restore the balance between bone formation and bone resorption in osteoporosis.
Notch signaling	DLL1, Jagged1, Notch receptors, TACE/ADAM17, gamma-secretase, NICD, CSL/CBF-1, HES/HEY family	Icariin modulates Notch signaling in a context-dependent manner and helps shift BMSC lineage commitment toward osteogenesis rather than adipogenesis.	Reduces adipogenic differentiation, increases ALP activity and osteogenic gene expression, and supports osteoblast lineage specification.	Helps prevent marrow adiposity and supports osteoblast differentiation in senescence- and estrogen deficiency-related osteoporosis.
BMP/Smad/Runx2/Osx signaling	BMP-2, BMP receptors, Smad1/5/8, Smad4, Runx2, Osterix, ALP, osteocalcin, type I collagen	Icariin activates BMP-associated osteogenic signaling, enhances Smad phosphorylation, and upregulates Runx2, Osterix, and other osteogenic markers.	Promotes osteoblastic differentiation of BMSCs, increases matrix mineralization, and accelerates bone formation and repair.	Counteracts impaired osteogenesis and strengthens the anabolic arm of bone remodeling in osteoporosis.
MAPK signaling	ERK1/2, p38 MAPK, JNK, ELK1, c-Myc, Runx2	Icariin fine-tunes MAPK signaling by activating ERK and p38 while suppressing excessive stress-associated signaling.	Enhances proliferation and osteogenic differentiation of BMSCs, increases ALP activity, and preserves osteogenic potential under pathological conditions.	Supports bone formation and protects BMSCs from dysfunction during osteoporosis progression.
OPG/RANK/RANKL signaling	OPG, RANK, RANKL, TRAF6, NF-kappaB, c-Fos, NFATc1, AP-1	Icariin increases the OPG/RANKL ratio and suppresses RANKL-mediated osteoclastogenic signaling.	Inhibits osteoclast differentiation and bone resorption while helping maintain coupling between osteoblasts and osteoclasts.	Reduces excessive bone resorption, preserves bone microarchitecture, and contributes to restoration of bone homeostasis in osteoporosis.

## LIST OF ABBREVIATIONS

ALP: Alkaline Phosphatase
BMSCs: Bone Marrow Mesenchymal Stem Cells
ICA: Icariin
OP: Osteoporosis
TCM: Traditional Chinese Medicine
